# Does Dietary Diversity Reduce the Risk of Obesity? Empirical Evidence from Rural School Children in China

**DOI:** 10.3390/ijerph17218122

**Published:** 2020-11-03

**Authors:** Chang Tao, Qiran Zhao, Thomas Glauben, Yanjun Ren

**Affiliations:** 1 College of Economics and Management, China Agricultural University, Beijing 100083, China; B20183110756@cau.edu.cn (C.T.); zhaoqiran@cau.edu.cn (Q.Z.); 2 Department of Agricultural Markets, Leibniz Institute of Agricultural Development in Transition Economies (IAMO), 06120 Halle (Saale), Germany; glauben@iamo.de

**Keywords:** dietary diversity, overweight, obesity, rural China

## Abstract

Childhood and adolescence overweight and obesity have implications for both health consequences and economic burden. Although it has been an emerging public health problem for primary school children in rural China and the importance of the diet–health link has been stressed for many years, rigorous analysis of the dietary diversity and obesity among children is rare. To clarify this issue, this study provides a better understanding of the functional linkage between dietary diversity and obesity by analyzing data from nearly 8500 rural primary students (aged from 10 to 13 years old) covering three provinces in China. Our estimation results show that there is a significantly negative correlation between dietary diversity and the probability of being overweight among primary students. In particular, for subgroups with higher dietary diversity, the negative correlation between dietary diversity and the incidence of overweight or obesity is stronger, and the absolute value of the coefficient is greater. The results also suggest that the increase in the consumption frequency of all dietary categories can significantly lead to a lower proportion of overweight. Thus, we conclude that higher dietary diversity can help to lower the risk of overweight and obesity among primary school children, presumably through increasing the daily frequency of food intakes and developing a more diverse dietary pattern.

## 1. Introduction

Childhood and adolescence overweight and obesity have implications for both present and future health and development, such as high blood pressure, early symptoms of hardening of the arteries, disordered breathing during sleep, and lifetime risk of being diagnosed with type 2 diabetes and obesity [1,2]. They also increase the double economic burden on the family and society, for example, the increase in state fiscal expenditure and the decrease in personal income [3,4].

Over the past 40 years, the prevalence of overweight and obesity among children has been rising worldwide [5]. In 2016, the global proportion of overweight and obesity among children aged 5–19 was as high as 18% (occurring similarly among 18% of girls and 19% of boys), which was just 4% in 1975 [6]. In the United States, according to the data provided by the Centers for Disease Control and Prevention (CDC), the obesity prevalence among children aged 2–19 was 18.5%; specifically, the prevalence was 13.9%, 18.4%, and 20.6% for children aged 2–5, 6–11 and 12–19, respectively [7]. In most of the European children aged 6–8, a higher proportion of boys are overweight and obese compared with girls; among them, the prevalence is highest in Mediterranean countries where nearly 50% of boys are overweight, and 20% of boys are obese [8]. However, most overweight and obese children live in developing countries, and with a growth rate of more than 30% higher than those in developed countries [5,6]. The number of overweight children under five in Asia is growing the fastest, accounting for almost half of the world in 2019; the overweight proportion of such population increased by nearly 24% during 2000–2019, in Africa [6,9]. In China, however, children bear a double burden of malnutrition, with the increasing burden of overweight and obesity along with the existing high burden of undernutrition [10,11], which has been a major public health problem in rural China. According to a 1% National Population Sample Survey 2015 in China, children aged 5–14 years represent 10.7 percent of China’s population (1374.6 million in 2015), 54.0 percent of whom lived in rural China [12]. According to *the Monitoring Report on the Nutrition and Health Status of Chinese Residents (2018)*, among children aged 6–17 in rural China, boys suffer from undernutrition at 17.3 percent and overweight/obese at 15.5 percent; for girls, it was 11.7 percent and 11.5 percent, respectively [13].

Many factors contribute to overweight and obesity among children, such as the family history of obesity [14], regular sugar-sweetened beverages [15], the involvement of grandparents in childcare [16], low levels of physical activity, high rate of sedentary leisure habits [17,18], dietary patterns [19], and various socioeconomic factors [20,21]. Dietary diversity is a proxy for nutrient adequacy of the diet of individuals [22]. Previous studies have shown the correlation between dietary diversity and the incidence of obesity, with conflicting results [23]. Although higher dietary diversity may be inversely related to obesity, due to the intake of more fiber, vitamins, and minerals [24], it may also lead to an increase in energy expenditure, which leads to excessive meat and calorie intake, and thus, increases in body mass index [25,26,27]. Besides, several school-based interventions have indicated that increased dietary diversity, especially improving with fruit and vegetable, contributed to the reduction of overweight and obesity in most groups of children and young adults [28,29,30,31]. Dietary diversity score is positively correlated with the nutritional adequacy status of children [32,33], and has been used by many recent studies on overweight and obesity issues [34,35]; the one chosen by us was especially in the contest of China [36,37]. Emerging literature has studied the relationship between children’s dietary diversity and various health conditions in China, such as micronutrient deficiencies [32], nutritional outcomes (measured by the incidence of stunting, underweight, and anemia) [38,39,40], and mental health problems [41]. Rigorous analysis of the dietary diversity score and obesity among children is rare in rural China.

This study provides a better understanding of the functional linkage between dietary diversity and obesity by analyzing data on nearly 8500 rural primary students (aged from 10–13) covering three provinces in China. Our estimation results indicate that there is a significantly negative correlation between dietary diversity and the probability of being overweight for pupils. Further, we also observe a significant heterogeneity effect of dietary diversity among subgroups dividing by dietary diversity score and personal and family characteristics. Specifically, dietary diversity is inversely associated with the incidence of overweight for students with a dietary diversity no less than four food groups, while it is inversely associated with the obesity ratio for students with a dietary diversity greater than six food groups. For subgroups with certain characteristics (such as female, less than 12 years old, having at least one sibling, and with low- and medium household assets), there is a negative correlation between dietary diversity and the incidence of overweight, while for students with at least one obese parent, increasing dietary diversity does not affect the incidence of overweight or obesity. We also explore the relationship between the intake frequency of food groups studied and the prevalence of overweight and obesity. Results suggest that, except for eggs, the increase in the frequency of daily food intakes can significantly reduce the proportion of overweight, and the increased frequency of consumption of tubers can significantly reduce the proportion of obesity. Thus, we can conclude that the higher dietary diversity can help to reduce the risk of overweight and obesity among primary school students, presumably through increasing the daily frequency of food intakes and developing a more diverse dietary pattern.

The remainder of the article proceeds as follows. Section 2 describes the methodology. Section 3 presents our findings, and Section 4 discusses its implications and limitations. Section 5 concludes the study.

## 2. Materials and Methods

### 2.1. Data

#### 2.1.1. Survey

This study uses a cross-sectional dataset that we collected in 2018 involving 8690 students (5th and 6th graders) in 90 rural primary schools from three provinces in China, namely, Henan Province, Anhui Province, and Yunnan Province. This study was approved by the Ethics Committee of China Agricultural University. All procedures performed in studies involving human participants were following the 1964 Helsinki declaration and its later amendments. All necessary permissions have also been obtained from the Chinese government and local education bureaus. All students participating in this survey and their caregivers, as well as their legal guardians in school (the principals and the headteachers), had a full understanding of the survey purpose and assented to participate in the project. All project participants were aware of the (minimal) risks involved and understood that their participation was entirely voluntary.

The Qinling–Huaihe Line, an important north–south geographical demarcation line in China, naturally divides the country into two regions; the northern and southern regions have formed many natural and human landscape differences, including dietary differences, over a long period of development [26,42]. We chose Henan Province as the representative of the northern region and Yunnan Province as the representative of the southern region, and Anhui Province was chosen because it was above the dividing line. According to the China Population and Employment Statistics Yearbook (2019), which is compiled by the Department of Population and Employment Statistics of the National Bureau of Statistics of China (NBSC-PESD) [9,12], Henan’s and Yunnan’s populations account for about 6.9% and 3.5% of the country’s population, respectively. Henan has been the second-most populous province in the northern region since 2010, and Yunnan is located in the middle of the southern region. Considering that China is a multi-ethnic country, the inclusion of Yunnan is also designed to cover the regions with ethnic minorities. Anhui is the only province that belongs to both the south and the north, and its population accounts for about 4.5% of the country’s population. Overall, the samples from Henan Province, Anhui Province, and Yunnan province accounted for 31.4%, 33.4%, and 35.3% of the entire sample, respectively.

#### 2.1.2. Sampling

The samples were collected using a multi-stage, stratified, random sampling procedure. First, for each province, we obtained a list of all of the counties and ranked them according to the local gross value of industrial output (GVIO) per capita of each county in 2016 [27,43]. Based on the list, we randomly chose five counties per province. Then we obtained a list of rural regular primary schools from each county’s bureau of education, leaving out those in the county seat (attended mostly by urban children) and the adult primary schools (attended by adults). Based on the school lists, we randomly selected six schools in each county. In total, we chose 30 schools in each province. In the next step, we randomly chose two Grade 5 and one Grade 6 classes per school, and all students in them became part of the sample. In total, we chose 202 classes (90% of the schools selected in our project had no more than two fifth-grade classes, so we chose the only one) and 2731 students in Henan, 2902 students in Anhui, and 3057 students in Yunnan.

### 2.2. Variables

The survey consisted of three main blocks. Specifically, all students completed a physical measurement (including height and weight), a 24-h dietary recall scale, and two questionnaires involving personal and family information.

#### 2.2.1. Outcomes Variables

In the first block of the survey, we measured 8453 sample students’ height and weight using standard instruments. Based on the physical measurement results, we calculated student body mass index-for-age z-score, using WHO AnthroPlus (Department of nutrition, World Health Organization, Geneva, Switzerland) and compared with reference data according to the WHO 2007 population. Although The body mass index (BMI = weight(kg)/height^2^(m^2^)) is widely used in adult populations [28,44], the BMI cut points that define obesity and overweight are not linked to age and sex. Children’s BMI cut points, however, vary with age and sex [29,45]. The body mass index-for-age z-score (based on The WHO Child Growth Standard) was developed to meet this condition and can be used in general practice for all children, irrespective of ethnicity [30,46]. Then we chose its cut points to define obesity and overweight, using values >+1 standard deviation(sd), >+2 sd, and <−2 sd to define overweight, obesity, and thinness, respectively; note that, in the estimation for overweight, we combine overweight (>+1 sd) with obesity (>+2 sd), taking the children (≤+1 sd but ≥−2 sd) as the reference group for a convenient interpretation.

**Figure 1:**
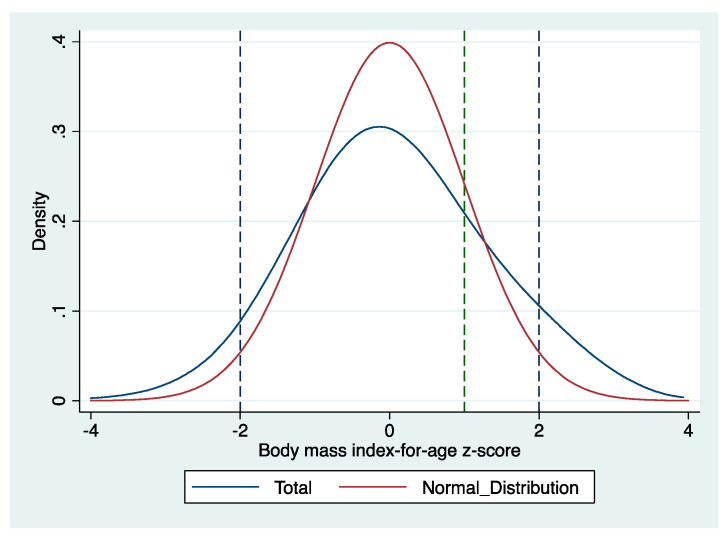
Distribution of body mass index-for-age z-score for entire sample and normal distribution (Mean = 0, Standard deviation = 1).

As shown in Table 1, the average body mass index-for-age z-score of the entire sample was 0.06. Over one-fifth of all students suffered from overweight (including obesity), and almost one-twentieth of all samples suffered from thinness. The rural primary school students bear the double burden of nutrition; that is, overweight/obesity and undernutrition coexist. Figure 1 shows the distribution of body mass index-for-age z-score, which is positioned to the right of the normed distribution. The distribution is also slightly flat, meaning that more students are obese and overweight, as well as thin.

#### 2.2.2. Dietary Diversity and Dietary Frequency

In the second block of the survey, each student filled out a 24-h dietary diversity recall scale on food consumption frequencies (but not actual amounts) of 33 food items, with possible help from trained enumerators and teachers (but not nutrition professionals). Before the formal investigation, we held a forum with the school administrative staff and parent representatives to find out the possible choices of students, which helps to ensure the quality of the diet recovery data. Based on the 24 h dietary recall data, we constructed a Dietary Diversity Score (DDS) to measure dietary diversity according to the guidelines provided by the Food and Agriculture Organization of the United Nations (FAO) [22]. Although the standard FAO guidelines cover 14 food groups (Table 2, column 2), due to data limitations, we aggregated some of them based on FAO guidelines and finally kept nine food groups for our evaluation (Table 2, column 1). Specifically, we calculated DDS by summing the number of food groups consumed by the student over the past 24 h. Those that the student had consumed over the 24-h recall period received a score of 1, otherwise a score of 0.

Terciles of dietary diversity was used to classify students into lowest, medium, and high dietary diversity by looking at the food groups consumed by at least 50% of students in each tertile [19,22], and the corresponding DDS is 0–3, 4–6, and 7–9, respectively, in this paper. In particular, the food groups included in the lowest tercile are “grains”, ”vegetables”, and ”meat”. “Tubers”, ”bean products, nuts, and seeds”, and “meat” were added by students in the medium tercile. All kinds of food items were consumed by at least 50% of students in the high tertile.

Since our data do not contain information on the specific amount of each food consumed, which is one limitation of the DDS, we used the 24 h recall information on the frequency of consuming food groups in each item as an alternative measure to approximate the students’ consumption level of each food group. During the 24-h recall period, students recalled whether each food group was consumed during breakfast, lunch, dinner, and snacks. The food item that the student had consumed each time received a score of 1, otherwise a score of 0. For example, if a student ate “meat” for lunch and dinner, his/her consumption frequency of “meat” is 2.

As presented in Table 3, the mean dietary diversity score of the total is 5.56. Four of the nine food groups were consumed more than once in 24 h, namely, “grains”, “vegetables”, “fruits”, and “meat”, of which “grains” were consumed most frequently at 1.34, while the consumption frequency of “fish” is the smallest, at 0.71.

#### 2.2.3. Control Variables

In the third survey block, students and their caregivers independently completed a questionnaire involving a series of personal and family level information. Based on these, we generated control variables, including personal characteristics (such as each student’s gender, age measured by month, and whether the student had received preschool education) and family characteristics (such as the number of siblings, parental age, education level, body mass index, and durable household assets) [11,14,17,20,23,31,39,47].

Of the total sample, 8388 students completed the entire survey. Note that due to the lack of information, the actual size of the analysis varies among different variables. For example, due to the absence of preschool education information, there are 8321 observations for the preschool dummy.

### 2.3. Estimation Method

#### 2.3.1. Association between Dietary Diversity and Nutrition Outcomes

The main objective of this study is to analyze the correlation between dietary diversity and the prevalence of being overweight and obese among primary school students, while controlling for observable individual and family characteristics. Multivariate regression analysis was performed in the following steps. First, we regressed DDS on the outcome variables and controlled the control variables mentioned in Section 2.2.3. Then, we add the county effect, because student’s dietary diversity may also be related to regional dietary differences. To gain a deeper understanding, we analyzed heterogeneity by subgroups (such as dietary diversity tercile, gender, and parental BMI), and explored the relationship between food groups’ (added to dietary pattern by students with higher DDS) intake frequency and the prevalence of overweight and obesity.

Since there are both continuous and binary outcome variables, we use different estimation methods. For the former, body mass index-for-age z-score, we first specify the ordinary least squared (OLS) model as follows:

(1) $$Outcomes_{ji}=\alpha +\beta DDS_{i}+\gamma X_{i}+\varepsilon_{ji},$$

where the outcome variable, $Outcomes_{ji}$, is the body mass index-for-age z-score or nutrition status (being overweight or obese) of student *i*. *DDS_i_* denotes the dietary diversity score of each one. The term β is the coefficient we are interested in, which measures the correlation between DDS and the incidence of overweight and obesity. The vector *X_i_* comprises a set of control variables (mentioned in Section 2.2.3) designed to capture part of the variation in outcomes, due to observable personal and family characteristics, and γ is the related coefficient vector. The term α is the intercept, and ε represents a random error that exists in a normal distribution. Here, *i* represents each of the observations.

The second model is similar to above, but in it, we add county effects:

(2) $$Outcomes_{jki}=\alpha +\beta DDS_{ki}+\gamma X_{ki}+Countyid_{k}+\varepsilon_{jki},$$

where $Outcomes_{jki}$ denotes the body mass index-for-age z-score or nutrition status (overweight or obesity) of student *i* in county *k*. *DDS* and all other control variables remain the same as in Equation (1). $Countyid_{k}$ represents a vector of county dummy to capture the county effects. The ordinary least squared (OLS) estimation is conducted for body mass index-for-age z-score, while the Probit estimation is applied for overweight and obesity.

#### 2.3.2. The Association between the Added Food Groups and Nutrition Outcomes

Furthermore, to analyze the correlation between the added food groups and the outcome variables, consider a statistical model:

(3) $$Outcomes_{jki}=\alpha +\beta_{p}Foodgroups_{kip}+\gamma X_{ki}+Countyid_{k}+\varepsilon_{jki},$$

where $Foodgroups_{kip}$ is the *p*th food group’s (e.g., “grains”, “tubers”, and so on) intake frequency of student *i* in county *k*, and all outcomes and control variables remain the same as in Equation (2). The food groups added by the students with a higher dietary diversity score include “tubers”, “bean products, nuts, and seeds”, “fish”, “fruits”, “eggs”, and “milk and milk products”. The estimation strategy is the same as for model (2).

## 3. Results

### 3.1. Correlation Analysis between Dietary Diversity Score and Obesity and Overweight

To analyze the correlation between Dietary Diversity Score (i.e., DDS) and obesity and overweight, as one of the main objectives of this paper, we estimate Equation (2) (with county fixed effects). As seen in Table 4, the results show a negative relationship between DDS and nutrition outcomes. Our findings indicate that, for every 1-point gain in DDS, the incidence of overweight significantly decreases by 0.005 (*p* < 0.10).

### 3.2. Heterogeneous Effects of Dietary Diversity on Obesity and Overweight

#### 3.2.1. Heterogeneity by Dietary Diversity Tercile

Since the incidence of obesity and overweight varies between the dietary diversity tercile subgroups (food groups consumed by ≥50% of students in each tercile), we used the same model as in Equation (2) to estimate the correlation between DDS and obesity and overweight to gain a deeper understanding of the correlation. The main results presented in Table 5 suggest that for subgroups with a dietary diversity of no less than four food groups (including the students with a DDS range from 4–9), a 1-point increase in DDS was associated with a 0.012 sd decrease in the overweight ratio (*p* <0.01). For subgroups with a dietary diversity greater than six food groups, a 1-point increase in DDS was associated with a 0.021 sd decrease in the overweight ratio (*p* <0.05), besides a 0.013 sd decreased in the obesity ratio (*p* < 0.01).

#### 3.2.2. Heterogeneity by Gender and Age

We also conducted additional regressions for the subgroups divided gender (boys or girls) and age (younger than 144 months old or not). The results in Table 6 suggest that for girls and younger students, increasing their dietary diversity can significantly reduce the risk of overweight. The regression results for girls indicate that the probability of being overweight is negatively correlated with dietary diversity score, with an absolute value of 0.006 sd. Besides this, DDS rises for students who are younger than 144 months (i.e., 12 years old), and the overweight ratio reduces by 0.007 sd.

#### 3.2.3. Heterogeneity by Family Characteristics

As seen in Table 7, for students with at least one sibling, a 1-point increase in DDS was associated with a 0.005 sd decrease in the overweight ratio (*p* < 0.10). For those whose father or mother was overweight (body mass index ≥ 25), a 1-point increase in DDS was associated with a 0.011 sd decrease in the overweight ratio (*p* < 0.05) and a 0.007 sd in the obesity ratio (*p* < 0.10). For those with assets scores (calculated by the principal components analysis) less than 0.5, a 1-point increase in DDS was associated with a 0.008 sd decrease in the overweight ratio (*p* < 0.01).

### 3.3. Correlation Analysis between Nutrition Outcomes and the Frequency of Six Main Food Groups

In addition to analyzing the correlation between mean dietary diversity scores and the incidence of obesity and overweight, it is also necessary to analyze which food group has a strong correlation with the incidence of obesity for students with higher dietary diversity scores. Since students with medium dietary diversity added two food groups (i.e., “tubers” and “bean products, nuts, and seeds”) to the main food consumed by the lowest tercile and those with high dietary diversity added four food groups (i.e., ”fishes”, “fruits”, ”eggs”, and ”milk and milk products”) to the main food consumed by the medium tercile, we estimated Equation (3) to analyze the correlation between the added food group consumption frequency and the incidence of obesity and overweight.

As seen in Table 8, except for eggs, the increase in the dietary frequency of all added food groups can significantly reduce the proportion of overweight, and increasing “tubers” consumption frequency can significantly reduce the proportion of obesity. Specifically, a 1-point increase in tuber consumption frequency was associated with a 0.018 sd and 0.014 sd decrease in the overweight ratio (*p* < 0.01) and obesity ratio (*p* < 0.01), respectively, and a 1-point increase in “bean products, nuts, and seeds”, “fish”, “fruit”, and “milk and milk products” consumption frequency was associated with a 0.023 sd (*p* < 0.01), 0.012 sd (*p* < 0.10), 0.012 (*p* < 0.10), and 0.020 (*p* < 0.01) decrease in the overweight ratio, respectively.

## 4. Discussion

Consistent with research conclusions based on a 2013 and earlier survey [13,48], rural Chinese children still face the double burden of malnutrition, the coexistence of under- and overnutrition. Compared with previous studies, as noted in the introduction [13], we found that the overall distribution of underweight is decreasing, while the proportion of overweight and obesity is increasing. This change is consistent with the national trends in thinness and overweight among Chinese school-aged children from 1985 to 2014 [49].

Previous studies in this field have shown conflicting results regarding the correlation between DDS and the incidence of obesity [24,26,27,32,50]; for example, there was an indirectly inverse association between the DDS and obesity in Isfahanian female university students [24], while a positive association between them was found for males in China [26] and preschool children in the US [27]. In others, no significant correlation was observed [32,50]. Conflicting results may be due to the use of different methods for assessing dietary intake and determination of DDS, the characteristics of the studied population, and differences in weight status assessment. In our sample, there was a negative correlation between DDS and the incidence of overweight. Although there is no reliable experimental evidence that increasing dietary diversity can reduce the risk of obesity in primary school children, limited diets were notably observed among stunted overweight children in China [51]. Our results provide a possible situation in a specific socio-cultural context, while targeting dietary diversity as an obesity prevention strategy in children requires more experimental results.

Interestingly, in the regression results divided by DDS levels, DDS is negatively correlated with the incidence of overweight in subsamples consuming greater than three food groups (i.e., DDS ≥ 4), and it is negatively correlated with the incidence of both overweight and obesity in subsamples consuming more than six food groups (i.e., DDS ≥ 7). This may be because the foods, added by those with a higher DDS, themselves help to reduce the risk of obesity, and they also squeeze out the consumption of high-fat and high-calorie foods. Studies have indicated that in most groups of children and young adults, increased food variety with more fruit and vegetable consumption was associated with lower energy consumption, and thus, contributed to the reduction of overweight and obesity [28,29,52,53] or at least helped maintain a normal weight [54]. In our sample, almost all of the students (over 99%) with a dietary diversity of no less than four food groups consumed vegetables; 74.39% of those with a dietary diversity of no less than seven food groups consumed fruit, which is significantly negatively correlated with the incidence of overweight.

Low whole-grain intake and high refined-grain intake were found to be the leading dietary risk factors in China [55]. It has been shown that bodyweight is positively associated with refined-grain intake [56]. Since tubers are generally regarded as substitutes for grains in China, tuber consumption may crowd out grain intake. In our sample, over 91.8% of the students with high dietary diversity consumed tubers, and tuber consumption frequency is negatively correlated with the incidence of both overweight and obesity.

In China, “bean products, nuts, and seeds” (especially bean products) are regarded as substitutes for meats, and increasing consumption of them often crowds out meat intake, and thus, decreases the consumption of high-fat foods. In our sample, over 96.6% of students consumed “bean products, nuts, and seeds”, the consumption frequency of which is negatively correlated with the incidence of overweight.

We also found that there is a significant inverse association between “milk and milk products” and the prevalence of overweight. This is consistent with some studies [57], but contrary to others [58]. The emergence of contradictory conclusions can be due to differences in sample characteristics and methods of nutritional status and dietary diversity assessment.

Although it has been shown that low Body Mass Index was associated with higher dietary diversity in young females but not in young males [24,53], there is not any significant correlation between them in our sub-regression results by gender. However, there is indeed a negative correlation between DDS and the incidence of overweight in girls.

Parental obesity is often considered an important risk factor for obesity in children [47,59]. We found that for the students whose father or mother was overweight, there was a significant negative correlation between DDS and their incidence of overweight and obesity; whereas, for those whose father or mother was obese, we could not find any significant correlation between DDS and the incidence of obesity or overweight. Since parents are responsible not only for passing onto their children their behaviors, but also for their genetic structure, for obese children whose parents are obese, it is difficult to improve their physical condition by adjusting their diet.

In contrast with existing research results [20,40], in this primary school student group, paternal education is positively correlated with the incidence of overweight and obese, while maternal education is negatively correlated with the incidence of overweight. It should be noted that rural China has been experiencing a large-scale out-migration to large cities for employment opportunities, which has resulted in a large number of children living apart from their parent(s). These children, called the left-behind children, accounted for 37.7% of total rural children and 21.9% of all children in China, nearly one third (32.7%) of whom are under the care of their grandparents, according to All-China Women’s Federation. Thus, for those, parental education may be more reflected in the family income, and the direct impact on the physical condition of children has been distorted. The grandparent generation, however, usually has lower educational attainment and poorer nutritional knowledge on healthy eating than parental caregivers in rural China [60]. Therefore, those children may be at higher risk of being overfed (by their grandparents). Future studies are needed to investigate caregiver differences within the specific socio-cultural contexts to shed light on the obesity risk in children and adolescents in rural China.

The target group of this study is primary school students (10 to 13 years old), who are in early adolescence and growing at a rapid pace [61]. To the best of our knowledge, studies focusing on the association between DDS and obesity in school-aged children in rural China are rare. Moreover, the conclusions of this paper provide materials for improving children and their caregivers’ nutritional cognition and references for improving children’s diet, and thus, improving their health. A more general implication is that to help build up a solid stock of human capital for China’s future labor force. The detailed analysis of heterogeneity provides a reference for the Chinese policymakers to specify implemented nutrition intervention programs more reasonably, to produce better human capital results.

However, when evaluating the results of this article, many limitations need to be considered. First, due to the cross-sectional sample, we cannot establish a causal association, but only analyze correlations. Second, this study was limited to children aged 10–13 years old and may not be generalizable to other age groups. Therefore, it will be beneficial to construct panel data and expand sample coverage in follow-up research and could provide more reliable and generalizable results. Despite this, since the study areas of this paper are located in rural China and are still low- and middle-income regions, the methodology of the analysis could be used by other researchers to carry out relevant research in low- and middle-income countries or regions. The analysis method in this article is suitable for most sample groups (including children or adults), but the specific variable construction method needs to be adjusted appropriately according to a certain sample.

## 5. Conclusions

This study provides a better understanding of the functional linkage between dietary diversity and obesity. Our estimation results show that there is a significantly negative correlation between dietary diversity and the probability of being overweight for primary students in rural China. To gain a deeper understanding of the correlation between DDS and obesity and overweight, we analyzed the differences among subgroups by dietary diversity tercile. Specifically, for subgroups with higher dietary diversity, the negative correlation between DDS and the incidence of overweight or obesity is stronger, and the absolute value of the coefficient is greater. We also conducted additional regressions for heterogeneity by personal and family characteristics. Students who are female, younger, have at least one sibling, have relatively few family assets, and whose parents are just overweight, but not obese, are more likely to reduce the incidence of overweight by increasing dietary diversity. Moreover, we estimated the correlation of six food groups (added by those with higher DDS) and the incidence of overweight and obesity. The results revealed that the higher dietary diversity leads to a lower risk of being overweight and obesity among primary school children, presumably through increasing food groups intake frequency per day and developing a more balanced dietary pattern, especially increasing the consumption of “tuber”, “fruit”, and “milk and milk products”.

## Figures and Tables

**Table 1:** Summary statistics of outcome variables.

Variable	Definition	Obs	Mean	Standard Deviation
**Outcome variables**				
BMI-for-age z-score (Baz)	Body mass index-for-age z-score	8388	0.06	1.24
Overweight	Dummy: =1 if yes (baz > +1 sd); =0 if normal (baz ≥ −2 sd, and baz ≤ +1 sd)	8041	0.23	0.42
Obesity	Dummy: =1 if yes (baz > +2 sd); =0 if normal (baz ≥ −2 sd, and baz ≤ +1 sd)	6797	0.09	0.28
Thinness	Dummy: =1 if yes (baz < −2 sd); =0 if normal (baz ≥ −2 sd, and baz ≤ +1 sd)	6548	0.05	0.22

Source: Authors’ survey.

**Table 2:** Comparisons of food categories involved in definitions of Dietary Diversity Score (DDS).

1. Food Groups Used to Construct DDS	2. Food Categories Involved in FAO Guidelines (FAO, 2008)
Grains	Grains
Tubers	Vitamin A-rich vegetables and tubers; white roots and tubers
Vegetables	Dark green leafy vegetables; other vegetables
Fruits	Vitamin A-rich fruits; other fruits
Meat	Flesh meat, organ meat
Eggs	Eggs
Fish	Fish and seafood
Bean products, nuts, and seeds	Legumes, nuts, and seeds
Milk and milk products	Milk and milk products
(no corresponding category)	Oil and fat

**Table 3:** Summary statistics of dietary diversity and dietary frequency within 24 h.

Variable	Definition	Obs	Mean	Standard Deviation
**Dietary diversity and dietary frequency within 24 h**				
Dietary Diversity Scores	The potential score range is 0–9.	8388	5.56	1.91
F_Grains	The potential dietary frequency range is 0–4	8388	1.34	0.54
F_Tubers	The potential dietary frequency range is 0–4	8388	0.99	0.65
F_Vegetables	The potential dietary frequency range is 0–4	8388	1.23	0.44
F_Fruits	The potential dietary frequency range is 0–4	8388	1.03	0.77
F_Bean products, nuts, and seeds	The potential dietary frequency range is 0–4	8388	0.96	0.55
F_Meat	The potential dietary frequency range is 0–4	8388	1.16	0.59
F_Fish	The potential dietary frequency range is 0–4	8388	0.71	0.73
F_Milk and milk products	The potential dietary frequency range is 0–4	8388	0.98	0.75
F_Eggs	The potential dietary frequency range is 0–4	8388	0.95	0.72

**Table 4:** Multivariate analysis of the correlation between DDS and nutrition outcomes for the overall sample.

Variables ^1^	1.	2.	3.
	Body Mass Index-for-Age z-Score	Overweight (=1 if Yes)	Obesity (=1 if Yes)
DDS	−0.010	−0.005 **	−0.002
	(0.007)	(0.003)	(0.002)
Boy (=1 if yes)	0.296 ***	0.106 ***	0.082 ***
	(0.025)	(0.009)	(0.007)
Agemonth (month)	−0.010 ***	−0.002 ***	−0.002 ***
	(0.001)	(0.000)	(0.000)
Preschool (=1 if yes)	0.007	0.022	0.004
	(0.043)	(0.017)	(0.013)
Sibling number	−0.097 ***	−0.030 ***	−0.026 ***
	(0.019)	(0.007)	(0.005)
Age_father (year)	−0.002	0.000	0.001
	(0.004)	(0.002)	(0.001)
Age_mother (year)	−0.008 *	−0.003 *	−0.001
	(0.004)	(0.002)	(0.001)
Edu_father (year)	0.010 **	0.004 **	0.002 *
	(0.005)	(0.002)	(0.001)
Edu_mother (year)	−0.007 *	−0.003 *	−0.001
	(0.004)	(0.002)	(0.001)
BMI_father	0.029 ***	0.009 ***	0.005 ***
	(0.004)	(0.001)	(0.001)
BMI_mother	0.042 ***	0.011 ***	0.007 ***
	(0.004)	(0.001)	(0.001)
Asset	0.033 ***	0.011 **	0.005
	(0.013)	(0.005)	(0.004)
County effects	Yes	Yes	Yes
Constant	0.294	-	-
	(0.382)	-	-
Observations	7975	7975	6743
R-squared/Chi2	0.090	382.79	319.56

(**^1^**) Robust standard errors in parentheses. *** *p* < 0.01, ** *p* < 0.05, * *p* < 0.1. Source: Authors’ survey.

**Table 5:** Heterogeneity in the correlation between DDS and nutrition outcomes by dietary diversity tercile.

Variables ^1^	1.	2.	3.
	Body Mass Index-for-Age z-Score	Overweight (=1 if Yes)	Obesity (=1 if Yes)
**Panel A: Subgroups**	Dietary diversity ≤ 3 food groups		
DDS	0.026	0.013	−0.006
	(0.043)	(0.015)	(0.011)
Controls	Yes	Yes	Yes
Observations	1201	1201	1023
R-squared/Chi2	0.120	88.05	89.7
**Panel B: Subgroups**	Dietary diversity ≥ 4 food groups		
DDS	−0.025 ***	−0.012 ***	−0.001
	(0.009)	(0.003)	(0.003)
Controls	Yes	Yes	Yes
Observations	6774	6774	5720
R-squared/Chi2	0.090	335.36	261.89
**Panel C: Subgroups**	Dietary diversity ≥ 7 food groups		
DDS	−0.066 **	−0.021 **	−0.013 *
	(0.028)	(0.010)	(0.008)
Controls	Yes	Yes	Yes
Observations	2630	2630	2261
R-squared/Chi2	0.112	162.47	130.04

^1^ Robust standard errors are in parentheses. *** *p* < 0.01, ** *p* < 0.05, * *p* < 0.1. Source: Authors’ survey.

**Table 6:** Heterogeneity in the correlation between DDS and nutrition outcomes by gender and age.

Variables ^1^	1.	2.	3.	4.	5.	6.
	Body Mass Index-for-Age z-Score	Over-Weight (=1 if Yes)	Obesity (=1 if Yes)	Body Mass Index-for-Age z-Score	Over-Weight (=1 if Yes)	Obesity (=1 if Yes)
**Panel A:** Gender	Boys			Girls		
DDS	−0.007	−0.005	−0.004	−0.013	−0.006 *	0.001
	(0.010)	(0.004)	(0.003)	(0.009)	(0.003)	(0.002)
Controls	Yes	Yes	Yes	Yes	Yes	Yes
Observations	4098	4098	3385	3877	3877	3358
R-squared/Chi2	0.084	170.8	149.16	0.077	136.22	84.31
**Panel B**: Age	Less than 144 months			At least 144 months		
DDS	−0.015	−0.007 *	−0.004	−0.002	−0.002	0.002
	(0.009)	(0.004)	(0.003)	(0.011)	(0.004)	(0.002)
Controls	Yes	Yes	Yes	Yes	Yes	Yes
Observations	4848	4848	4072	3127	3127	2671
R-squared/Chi2	0.101	273.34	210.78	0.066	119.67	122.9

^1^ Robust standard errors are in parentheses. *** *p* < 0.01, ** *p* < 0.05, * *p* < 0.1. Source: Authors’ survey.

**Table 7:** Heterogeneity in the correlation between DDS and nutrition outcomes by family characteristics.

Variables ^1^	1.	2.	3.	4.	5.	6.
	Body Mass Index-for-Age z-Score	Over-Weight (=1 if Yes)	Obesity (=1 if Yes)	Body Mass Index-for-Age z-Score	Over-Weight (=1 if Yes)	Obesity (=1 if Yes)
**Panel A**: Sibling number	At least 1 sibling			Only child		
DDS	−0.010	−0.005 *	−0.002	−0.010	−0.010	−0.001
	(0.007)	(0.003)	(0.002)	(0.023)	(0.008)	(0.006)
Controls	Yes	Yes	Yes	Yes	Yes	Yes
Observations	7153	7153	6052	822	822	691
R-squared/Chi2	0.092	335.96	297.26	0.088	57.99	34.68
**Panel B**: Parents’ BMI	BMI_father ≥ 25 or BMI_mother ≥ 25			BMI_father ≥ 30 or BMI_mother ≥ 30		
DDS	−0.021 *	−0.011 **	−0.007 *	0.008	−0.002	−0.006
	(0.012)	(0.005)	(0.004)	(0.028)	(0.012)	(0.011)
Observations	2864	2864	2312	457	457	370
Controls	Yes	Yes	Yes	Yes	Yes	Yes
R-squared/Chi2	0.084	142.79	138.73	0.135	55.99	55.38
**Panel C**: Household assets	Low- and medium (<0.5)			High (>0.5)		
DDS	−0.016 *	−0.008 ***	−0.003	0.001	−0.000	0.002
	(0.009)	(0.003)	(0.002)	(0.011)	(0.004)	(0.003)
Controls	Yes	Yes	Yes	Yes	Yes	Yes
Observations	4773	4773	4070	3202	3202	2673
R-squared/Chi2	0.094	226.94	216.41	0.086	175.05	125.74

^1^ Robust standard errors are in parentheses. *** *p* < 0.01, ** *p* < 0.05, * *p* < 0.1. Source: Authors’ survey.

**Table 8:** The correlation between nutrition outcomes and the frequency of six main food groups.

Estimation	Variables ^1^	1.	2.	3.
		Body Mass Index-for-Age z-Score	Overweight (=1 if Yes)	Obesity (=1 if Yes)
1.	F_Tubers	−0.045 **	−0.018 ***	−0.014 ***
		(0.019)	(0.007)	(0.005)
2.	F_Bean products, nuts and seeds	−0.051 **	−0.023 ***	−0.007
		(0.022)	(0.009)	(0.006)
3.	F_Fish	−0.038 **	−0.012 *	−0.002
		(0.018)	(0.007)	(0.005)
4.	F_Fruits	−0.030 **	−0.012 *	−0.005
		(0.016)	(0.006)	(0.004)
5.	F_Eggs	−0.026	−0.007	−0.001
		(0.017)	(0.007)	(0.005)
6.	F_Milk and milk products	−0.048 ***	−0.020 ***	−0.007
		(0.016)	(0.006)	(0.005)
	Observations	7975	7975	6743
	R-squared/Chi2	0.090	380.86	319.66

^1^ Robust standard errors are in parentheses. *** *p* < 0.01, ** *p* < 0.05, * *p* < 0.1. The control variables for each estimation are identical to controls in Table 3. Source: Authors’ survey.

## References

[1] Daniels S.R. (2006). The consequences of childhood overweight and obesity. Future Child..

[2] Singh A.S., Mulder C., Twisk J.W., Van Mechelen W., Chinapaw M.J. (2008). Tracking of childhood overweight into adulthood: A systematic review of the literature. Obes. Rev..

[3] Liu X., Zhu C. (2014). Will knowing diabetes affect labor income? Evidence from a natural experiment. Econ. Lett..

[4] Su W., Huang J., Chen F., Iacobucci W., Mocarski M., Dall T., Perreault L. (2015). Modeling the clinical and economic implications of obesity using microsimulation. J. Med. Econ..

[5] IFPRI (2016). Global Nutrition Report 2016: From Promise to Impact: Ending Malnutrition by 2030.

[6] WHO (2016). World Health Organization Obesity and Overweight Fact Sheet.

[7] Hales C.M., Carroll M.D., Fryar C.D., Ogden C.L. (2017). Prevalence of Obesity among Adults and Youth: United States, 2015–2016.

[8] WHO (2019). World Health Statistics Overview 2019: Monitoring Health for the SDGs, Sustainable Development Goals.

[9] WHO (2016). Consideration of the Evidence on Childhood Obesity for the Commission on Ending Childhood Obesity: Report of the *Ad hoc* Working Group on Science and Evidence for Ending Childhood Obesity.

[10] Wang Y., Monteiro C., Popkin B.M. (2002). Trends of obesity and underweight in older children and adolescents in the United States, Brazil, China, and Russia. Am. J. Clin. Nutr..

[11] Zhang N., Ma G. (2018). Childhood obesity in China: Trends, risk factors, policies and actions. Glob. Health J..

[12] Department of Population and Employment Statistics of the National Bureau of Statistics of China (2019). China Population and Employment Statistics Yearbook-2019.

[13] Zhang Q., Hu X. (2018). Monitoring Report on Nutrition and Health Status of Chinese Residents No. 11: 2010–2013 Nutrition and Health Status of Chinese School Children Aged 6–17.

[14] He Q., Ding Z., Fong D., Karlberg J. (2000). Risk factors of obesity in preschool children in China: A population-based case–control study. Int. J. Obes..

[15] Shang X.W., Liu A.L., Zhang Q., Hu X.Q., Du S.M., Jun M., Xu G.F., Ying L., Guo H.W., Lin D. (2012). Report on childhood obesity in China (9): Sugar-sweetened beverages consumption and obesity. Biomed. Environ. Sci..

[16] Li B., Adab P., Cheng K.K. (2015). The role of grandparents in childhood obesity in China-evidence from a mixed methods study. Int. J. Behav. Nutr. Phys. Act..

[17] Schmitz K.H., Lytle L.A., Phillips G.A., Murray D.M., Birnbaum A.S., Kubik M.Y. (2002). Psychosocial correlates of physical activity and sedentary leisure habits in young adolescents: The Teens Eating for Energy and Nutrition at School study. Prev. Med..

[18] Li M., Xue H., Wen M., Wang W., Wang Y. (2017). Nutrition and physical activity related school environment/policy factors and child obesity in China: A nationally representative study of 8573 students in 110 middle schools. Pediatric Obes..

[19] Zhang J., Wang H., Wang Y., Xue H., Wang Z., Du W., Su C., Zhang J., Jiang H., Zhai F. (2015). Dietary patterns and their associations with childhood obesity in China. Br. J. Nutr..

[20] Liu W., Liu W., Lin R., Li B., Pallan M., Cheng K., Adab P. (2016). Socioeconomic determinants of childhood obesity among primary school children in Guangzhou, China. BMC Public Health.

[21] Tamayo T., Herder C., Rathmann W. (2010). Impact of early psychosocial factors (childhood socioeconomic factors and adversities) on future risk of type 2 diabetes, metabolic disturbances and obesity: A systematic review. BMC Public Health.

[22] Kennedy G., Ballard T., Dop M.C. (2011). Guidelines for Measuring Household and Individual Dietary Diversity.

[23] Salehi-Abargouei A., Akbari F., Bellissimo N., Azadbakht L. (2016). Dietary diversity score and obesity: A systematic review and meta-analysis of observational studies. Eur. J. Clin. Nutr..

[24] Azadbakht L., Esmaillzadeh A. (2012). Dietary energy density is favorably associated with dietary diversity score among female university students in Isfahan. Nutrition.

[25] Jayawardena R., Byrne N.M., Soares M.J., Katulanda P., Yadav B., Hills A.P. (2013). High dietary diversity is associated with obesity in Sri Lankan adults: An evaluation of three dietary scores. BMC Public Health.

[26] Tian X., Wu M., Zang J., Zhu Y., Wang H. (2017). Dietary diversity and adiposity in Chinese men and women: An analysis of four waves of cross-sectional survey data. Eur. J. Clin. Nutr..

[27] Fernandez C., Kasper N.M., Miller A.L., Lumeng J.C., Peterson K.E. (2016). Association of dietary variety and diversity with body mass index in US preschool children. Pediatrics.

[28] Rolls B.J., Ello-Martin J.A., Tohill B.C. (2004). What can intervention studies tell us about the relationship between fruit and vegetable consumption and weight management?. Nutr. Rev..

[29] Spiegel S.A., Foulk D. (2006). Reducing overweight through a multidisciplinary school-based intervention. Obesity.

[30] Xu H., Ecker O., Zhang Q., Du S., Liu A., Li Y., Hu X., Li T., Guo H., Li Y. (2020). The effect of comprehensive intervention for childhood obesity on dietary diversity among younger children: Evidence from a school-based randomized controlled trial in China. PLoS ONE.

[31] Foster G.D., Sherman S., Borradaile K.E., Grundy K.M., Vander Veur S.S., Nachmani J., Karpyn A., Kumanyika S., Shults J. (2008). A policy-based school intervention to prevent overweight and obesity. Pediatrics.

[32] Zhao W., Yu K., Tan S., Zheng Y., Zhao A., Wang P., Zhang Y. (2017). Dietary diversity scores: An indicator of micronutrient inadequacy instead of obesity for Chinese children. BMC Public Health.

[33] Arimond M., Ruel M.T. (2004). Dietary diversity is associated with child nutritional status: Evidence from 11 demographic and health surveys. J. Nutr..

[34] Hooshmand S., Udipi S.A. (2013). Dietary diversity and nutritional status of urban primary school children from Iran and India. J. Nutr. Disord. Ther..

[35] Karimbeiki R., Pourmasoumi M., Feizi A., Abbasi B., Hadi A., Rafie N., Safavi S. (2018). Higher dietary diversity score is associated with obesity: A case–control study. Public Health.

[36] Bi J., Liu C., Li S., He Z., Chen K., Luo R., Wang Z., Yu Y., Xu H. (2019). Dietary diversity among preschoolers: A cross-sectional study in poor, rural, and ethnic minority areas of central south china. Nutrients.

[37] Yang Y.X., Wang X.L., Leong P.M., Zhang H.M., Yang X.G., Kong L.Z., Zhai F.Y., Cheng Y.Y., Guo J.S., Su Y.X. (2018). New Chinese dietary guidelines: Healthy eating patterns and food-based dietary recommendations. Asia Pac. J. Clin. Nutr..

[38] Wang A., Scherpbier R.W., Huang X., Guo S., Yang Y., Josephs-Spaulding J., Ma C., Zhou H., Wang Y. (2017). The dietary diversity and stunting prevalence in minority children under 3 years old: A cross-sectional study in forty-two counties of Western China. Br. J. Nutr..

[39] Chen Q., Pei C., Zhao Q. (2018). Eating More but Not Better at School? Impacts of Boarding on Students’ Dietary Structure and Nutritional Status in Rural Northwestern China. Sustainability.

[40] Chen Q., Pei C., Bai Y., Zhao Q. (2019). Impacts of Nutrition Subsidies on Diet Diversity and Nutritional Outcomes of Primary School Students in Rural Northwestern China-Do Policy Targets and Incentives Matter?. Int. J. Environ. Res. Public Health.

[41] Liu X., Zhao Q., Chen Q. (2019). Better nutrition, healthier mind? Experimental evidence from primary schools in rural northwestern China. J. Integr. Agric..

[42] Liu J., Yang Q., Liu J., Zhang Y., Jiang X., Yang Y. (2020). Study on the Spatial Differentiation of the Populations on Both Sides of the “Qinling-Huaihe Line” in China. Sustainability.

[43] Rozelle S. (1996). Stagnation without equity: Patterns of growth and inequality in China’s rural economy. China J..

[44] WHO (1995). Physical Status: The Use of and Interpretation of Anthropometry, Report of a WHO Expert Committee.

[45] Must A., Anderson S. (2006). Body mass index in children and adolescents: Considerations for population-based applications. Int. J. Obes..

[46] Onis M.d., Onyango A.W., Borghi E., Siyam A., Nishida C., Siekmann J. (2007). Development of a WHO growth reference for school-aged children and adolescents. Bull. World Health Organ..

[47] Lazzeri G., Pammolli A., Pilato V., Giacchi M.V. (2011). Relationship between 8/9-yr-old school children BMI, parents’ BMI and educational level: A cross sectional survey. Nutr. J..

[48] Zhou S., Ye B., Fu P., Li S., Yuan P., Yang L., Zhan X., Chao F., Zhang S., Wang M.Q. (2020). Double Burden of Malnutrition: Examining the Growth Profile and Coexistence of Undernutrition, Overweight, and Obesity among School-Aged Children and Adolescents in Urban and Rural Counties in Henan Province, China. J. Obes..

[49] Song Y., Agardh A., Ma J., Li L., Lei Y., Stafford R.S., Prochaska J.J. (2019). National trends in stunting, thinness and overweight among Chinese school-aged children, 1985–2014. Int. J. Obes..

[50] Amirhamidi Z., Omidvar N., Eini-Zinab H., Doustmohammadian A., Esfandiari S., Azadi R., Haidari H. (2019). Association of Weight Status with Dietary Intake and Dietary Diversity Score in 10–12-Year-Old Children in Tehran: A Cross-Sectional Study. Iran. J. Pediatrics.

[51] Li Y., Wedick N.M., Lai J., He Y., Hu X., Liu A., Du S., Zhang J., Yang X., Chen C. (2011). Lack of dietary diversity and dyslipidaemia among stunted overweight children: The 2002 China National Nutrition and Health Survey. Public Health Nutr..

[52] Kennedy E., Powell R. (1997). Changing eating patterns of American children: A view from 1996. J. Am. Coll. Nutr..

[53] McCrory M.A., Fuss P.J., McCallum J.E., Yao M., Vinken A.G., Hays N.P., Roberts S.B. (1999). Dietary variety within food groups: Association with energy intake and body fatness in men and women. Am. J. Clin. Nutr..

[54] Natale R.A., Lopez-Mitnik G., Uhlhorn S.B., Asfour L., Messiah S.E. (2014). Effect of a child care center-based obesity prevention program on body mass index and nutrition practices among preschool-aged children. Health Promot. Pract..

[55] Li Y., Wang D.D., Ley S.H., Vasanti M., Howard A.G., He Y., Hu F.B. (2017). Time trends of dietary and lifestyle factors and their potential impact on diabetes burden in China. Diabetes Care.

[56] Liu S., Willett W.C., Manson J.E., Hu F.B., Rosner B., Colditz G. (2003). Relation between changes in intakes of dietary fiber and grain products and changes in weight and development of obesity among middle-aged women. Am. J. Clin. Nutr..

[57] Motamed Rezaei O., Moodi M., Tiyuri A., ZarShenas F., Sharifi M. (2016). Prevalence of obesity and its relationship with food habits among 10–14 years old school boys in Birjand, 2014: A short report. J. Rafsanjan Univ. Med. Sci..

[58] Pei Z., Flexeder C., Fuertes E., Standl M., Buyken A., Berdel D., Von Berg A., Lehmann I., Schaaf B., Heinrich J. (2014). Food intake and overweight in school-aged children in Germany: Results of the GINIplus and LISAplus studies. Ann. Nutr. Metab..

[59] Maffeis C., Talamini G., Tato L. (1998). Influence of diet, physical activity and parents’ obesity on children’s adiposity: A four-year longitudinal study. Int. J. Obes..

[60] Zhang N., Bécares L., Chandola T., Callery P. (2015). Intergenerational differences in beliefs about healthy eating among carers of left-behind children in rural China: A qualitative study. Appetite.

[61] Das J.K., Salam R.A., Thornburg K.L., Prentice A.M., Campisi S., Lassi Z.S., Koletzko B., Bhutta Z.A. (2017). Nutrition in adolescents: Physiology, metabolism, and nutritional needs. Ann. N. Y. Acad. Sci..

